# Influence of lung function on macro- and micro-structural brain changes in mid- and late-life

**DOI:** 10.1097/JS9.0000000000002228

**Published:** 2025-01-24

**Authors:** Jiao Wang, Weige Xu, Abigail Dove, Alireza Salami, Wenzhe Yang, Xiangyu Ma, David A. Bennett, Weili Xu

**Affiliations:** a Department of Epidemiology, College of Preventive Medicine,Third Military Medical University, Chongqing, China; b Department of Radiology, Tianjin Gongan Hospital, Tianjin, China; c Aging Research Center, Department of Neurobiology, Care Sciences and Society, Karolinska Institutet, Stockholm, Sweden; d Department of Epidemiology and Biostatistics, School of Public Health, Tianjin Medical University, Tianjin, China; e Rush Alzheimer’s Disease Center, Rush University Medical Center, Chicago, Illinois

**Keywords:** diffusion, lung function, magnetic resonance imaging, UK Biobank, white matter microstructure

## Abstract

**Introduction:**

Lung function has been associated with cognitive decline and dementia, but the extent to which lung function impacts brain structural changes remains unclear. We aimed to investigate the association of lung function with structural macro- and micro-brain changes across mid- and late-life.

**Methods:**

The study included a total of 37 164 neurologic disorder-free participants aged 40–70 years from the UK Biobank, who underwent brain MRI scans 9 years after baseline. After 2.5 years, a subsample (n = 3895) underwent a second MRI scan. Lung function was assessed using a composite score based on forced expiratory volume in 1 second, forced vital capacity, and peak expiratory flow, and divided into tertiles (i.e., low, moderate, and high). Structural brain volumes (including total brain, gray matter, white matter, hippocampus, and white matter hyperintensities) and diffusion markers (fractional anisotropy [FA] and mean diffusivity [MD]) were assessed. Data were analyzed using linear regression and mixed-effects models.

**Results:**

Compared to high lung function, low lung function was associated with smaller total brain, gray matter, white matter, and hippocampal volume, as well as lower white matter integrity. Over the 2.5-year follow-up, low lung function was associated with reduced white matter and hippocampal volume, reduced FA, and increased white matter hyperintensity volume and MD. After stratification by age, the associations remained significant among adults aged 40–60 years and 60+ years.

**Conclusion:**

Low lung function is associated with macro- and micro-structural brain changes involving both neurodegenerative and vascular pathologies. This association is significant in both mid- and late-life.

## Introduction

Lung function, a predictor of death and disability in older adults^[1,2]^, tends to decline during the aging process due to changes in elastic recoil and chest compliance^[3]^. Lung function can be assessed by several measures of ventilatory function, such as forced expiratory volume in 1s (FEV1), forced vital capacity (FVC), and peak expiratory flow (PEF), as well as FEV1/FVC^[4]^. Poor lung function is associated with chronic brain hypoxia^[5]^, which may further contribute to cognitive decline and increased risk of dementia^[6,7]^.

Several studies have shown that poor lung function is associated with cognitive decline and dementia^[6-10]^, however, the mechanisms behind this association remain unclear. Brain magnetic resonance imaging (MRI) is an important tool for understanding brain structure, offering an opportunity to evaluate the possible mechanisms underlying cognitive changes^[11,12]^. In addition, MRI can perform functional measurements and diffusion tensor imaging (DTI), providing the basis for the measurement of brain microstructure^[13,14]^. Poor lung function may be linked to a range of cerebral hypoxic responses, which in turn may lead to neurodegenerative and vascular lesions in the brain, including total brain, gray matter [as well as regional gray matter], white matter, and hippocampus, white matter hyperintensity (WMH), and white matter integrity, via cerebral vascular perfusion, ischemia, oxidative stress, and inflammation^[15]^. Only a few clinical studies have examined the relationship between lung function and structural brain volumes, and with inconsistent results^[7,16-19]^. Some linked poor lung function with brain atrophy and greater WMH^[7,16,19]^, while others found no such association^[17,18]^. To date, no large population-based studies have investigated the longitudinal association of lung function with macro- and micro-structural brain changes.

Previous evidence has shown that accelerated physiological aging of the lungs begins at the age of about 50–55 years and is associated with subsequent health conditions^[20,21]^. Meanwhile, some studies suggest that mid-life lung function is also associated with cognitive decline and dementia^[22,23]^. Most studies on the association between lung function and structural brain differences have examined only older adults^[7,16,17]^, so the impact of poor lung function on the brain in mid-life is unclear.

We hypothesize that poor lung function is associated with brain aging in mid- and late-life. In the present study, we aimed to verify this hypothesis by (1) examining the association of lung function with macro- and micro-structural brain changes on MRI; and (2) exploring whether this association is present in both mid- and late-life using data from a large population-based neuroimaging study within the UK Biobank.

## Methods and materials

### Study design, setting, and participants

Data were derived from the UK Biobank, a large-scale population-based cohort study including 502 412 UK residents aged 37–73 years^[24]^. The baseline survey started in 2006, with the first follow-up in 2012, the second follow-up imitated in 2014, and the third follow-up examination beginning in 2019.

**Figure 1.**
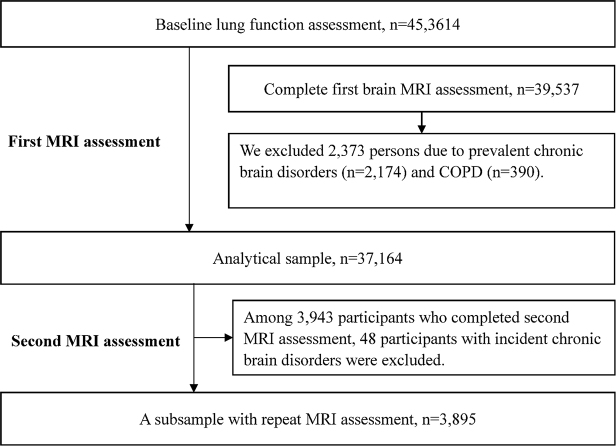
Flowchart of participants included in the study. Abbreviation: MRI = magnetic resonance imaging; COPD = chronic obstructive pulmonary disease.

Of all the participants, 453 614 underwent an assessment for lung function at baseline, of whom 39 537 also underwent MRI assessment in the second follow-up. From this group, in order to avoid the presence of reverse causation, we excluded 2373 participants, including those with prevalent chronic brain disorders (n = 2174; Supplementary Table 1, available at: http://links.lww.com/JS9/D766) or chronic obstructive pulmonary disease (COPD, n = 390) identified through combined information from self-report and medical registry, leaving a total of 37 164 participants for the cross-sectional analysis (Fig. 1). The longitudinal analysis was restricted to the 3895 participants who underwent a second MRI assessment during the third follow-up (Fig. 1).

### Ethics statement

The UK Biobank study received ethical approval from the National Health Services (NHS) National Research Ethics Service (Ref 11/NW/0382). All enrolled participants provided informed and written consent.

### Assessment of lung function

Lung function was tested at baseline using a Vitalograph Pneumotrac 6800 spirometer (Maids Moreton, UK) measuring forced vital capacity (FVC, the maximum amount of air that can be exhaled), forced expiratory volume in 1 s (FEV1, the amount of air that can be exhaled in one second), and peak expiratory flow (PEF, the maximum speed of expiration)^[25]^.

Two blows were recorded for each participant and a third blow was performed if there was a >5% difference between the FVC and FEV1 measures in the first two blows. We calculated the average of all available readings. Next, the raw scores of the averaged FVC, FEV1, and PEF component measures were converted into z-scores using the means and standard deviations calculated from the entire study sample at baseline. Finally, a comprehensive lung function score was created by averaging these z-scores. The composite lung function score provides a more comprehensive and sensitive reflection of pulmonary dysfunction and has been validated in previous studies^[7,26]^. Given that these measures are affected by several factors unrelated to pulmonary function (such as effort)^[27]^, we also calculated the FEV1/FVC ratio using the best FEV1 and FVC values taken from each participant’s consecutive blows. FEV1% predicted was calculated using the Global Lung Initiative (GLI) formula standardized by age, sex, race, and height.

The composite lung function index was operationalized as both a continuous and a categorical variable. Based on previous studies and the distribution of lung function^[7]^, intervals for lung function were derived from sex-specific tertiles (i.e., low, moderate, and high [as reference]), minimizing the confounding effect of sex on lung function and resulting in approximately equally sized subgroups.

### MRI data acquisition and pre-processing

Details of the image acquisition and processing are available on the UK Biobank website in the brain scan protocol and brain imaging documentation^[28,29]^. Briefly, participants underwent MRI scans at one of four centers including Cheadle (58.68%), Reading (15.43%), Newcastle (25.75%), and Bristol (0.14%). All centers used a Siemens Skyra 3 T scanner with a standard Siemens 32-channel head coil. T1-weighted imaging (resolution: 1.0 × 1.0 × 1.0 mm; field-of-view: 208 × 256 × 256 matrix) and T2 FLAIR imaging (resolution: 1.05 × 1.0 × 1.0 mm; field-of-view: 192 × 256 × 256 matrix) were performed to provide volumes of brain tissues and structures. DTI was applied to estimate the diffusion properties reflecting the integrity of microstructural tissue compartments, and 36 slices (resolution: 2.0 × 2.0 × 2.0 mm; field-of-view:104 × 104 × 72 matrix) were obtained using an echo plane, single-shot Stejskal-Tanner pulse sequence (echo time: 92 ms) in 50 distinct diffusion-weighted directions (b = 1000 and 2000 sec/mm^2^). Eigenvectors, eigenvalues, and fractional anisotropy (FA) were calculated by feeding the b value of 1000 sec/mm^2^ shell into the DTI fitting tool (DTIFIT version 2.0; the FSL diffusion tensor fitting program, FMRIB), which generated the FA and mean diffusivity (MD) outputs (Supplementary Methods 2, available at: http://links.lww.com/JS9/D766).

Summary measures of macro- and micro-brain structure have been generated by an image-processing pipeline developed and run on behalf of the UK Biobank, using publicly available image processing tools FSL (the FMRIB Software Library, version 5.0.10, http://fsl.fmrib.ox.ac.uk/fsl) and FreeSurfer (version 6.0)^[30]^. In this study, the volumes (in cubic millimeters) of total brain, gray matter, white matter, regional gray matter volumes (i.e., volumes of gray matter in the superior frontal gyrus, inferior frontal gyrus, middle frontal gyrus, supplementary motor cortex, precentral gyrus, postcentral gyrus, precuneus, superior parietal lobe, parahippocampal gyrus, middle temporal gyrus, and inferior temporal gyrus), hippocampus, and white matter hyperintensities (WMH) were assessed. Tract-averaged FA and MD values were acquired from the 27 white matter tracts for association fibers, commissural fibers, and projection fibers (Supplementary Figures 1 and 2, available at: http://links.lww.com/JS9/D766)^[31]^. All MRI parameters were converted to z-scores, and WMH volume was log-transformed due to skewed distribution. In this study, structural brain volumes were measured, including total brain, gray matter, white matter, and hippocampus volumes. White matter integrity was assessed using FA, MD, and WMH.

### Assessment of covariates

Questionnaire-based data were used for the following (Supplementary Method 1 and Supplementary Table 2, available at: http://links.lww.com/JS9/D766): sex, race (white or non-white [including Asian or Asian British, Black or Black British, and Mixed]), Townsend deprivation index (TDI), education (college or non-college), body mass index (BMI), smoking and alcohol use status (i.e., never, former, or current), smoking pack years (number of cigarettes per day/20 × [age stopped smoking − age start smoking]), regular physical activity, high level of social connection (≥once a week), diabetes, hypertension, heart disease (including myocardial infarction, angina, congestive heart failure, and atrial fibrillation), and respiratory disease (including respiratory infection [pneumonia, lung abscess, and empyema], asthma, and allergies). Moreover, the *apolipoprotein E* (*APOE* ɛ4), is strongly associated with Alzheimer’s disease and dementia. In this study, *APOE* was genotyped into ɛ4 alle carriers and non-ɛ4 carriers based on blood samples collected at baseline^[32]^.

### Statistical analysis

We used chi-square tests (categorical variables) and one-way ANOVA/Wilcoxon rank-sum tests (normal/non-normal continuous variables) to compare the baseline characteristics of the study population by lung function categories.

The latent measures of global white matter fractional anisotropy (gFA) and mean diffusivity (gMD) were derived using confirmatory factor analysis to address the high degree of covariance between white matter microstructural properties across the brain^[31,33]^. Extreme outlying data (further than ±4 standard deviations from the mean) were excluded to avoid potential measurement bias. Then, major white matter tracts were used for FA (n = 34 228 at baseline including 3796 having repeated measurements) and MD (n = 34 391 at baseline including 3807 having repeated measurements) modelling. All 27 major white matter tracts (including both the left and right hemisphere structures) were entered into the model separately, and the correlation residuals between other tracts were allowed. The medial lemniscus (L + R), middle cerebellar peduncle, and parahippocampal part of cingulum (L + R) were not included in the final factor analysis due to their low loading (i.e., factor loading <0.3). The confirmatory factor analysis was performed twice in both the first and repeated MRI measures and was consistent with longitudinal measurement invariance. Factor loadings and model fit metrics (CFI > 0.9, NFI > 0.9, RMSEA < 0.08) are shown in Supplementary Table 3, available at: http://links.lww.com/JS9/D766.

In the cross-sectional analysis (i.e., association between baseline lung function and MRI measures assessed at the second follow-up period), linear regression models were used to estimate the β-coefficients and 95% confidence intervals (CIs) for the relationship between baseline lung function (three individual lung function measures, FEV1/FVC, and the composite measure; as a continuous and a categorical variable) and MRI parameters. In the longitudinal analysis (i.e., association between baseline lung function and repeated MRI measures assessed at the second and third follow-up period), linear mixed-effects models were used to estimate the β-coefficients and 95% CIs for the associations between lung function with changes in brain structure. The fixed effect included lung function, follow-up time, and their interaction. The random effect included random intercept and slope, allowing the individual differences at baseline and across follow-up. Models (both cross-sectional and longitudinal analysis) were adjusted for age, sex, education, TDI, BMI, alcohol consumption, smoking, physical activity, social connection, hypertension, diabetes, heart disease, *APOE* ε4 carrier status, and MRI head position (volumetric data were also adjusted for the volumetric scaling from T1 head image to standard space). We also performed stratified analysis by age group (mid-life [<60 years] and late life [≥60 years]). Statistical interaction was examined by creating a binary indicator variable with the cross-product of age (<60 years vs. ≥60 years) and lung function (continuous) in the models to test for statistical significance.

In sensitivity analysis, we repeated the analyses after (1) performing multiple imputation for missing values of lung function or other covariates (n = 1634); (2) further adjusting for MRI assessment center; (3) further adjusting for respiratory disease; (4) using FEV1% predicted as lung function indicator; (5) further adjusting for smoking pack years to eliminate the cumulative impact of tobacco smoking; and (6) further adjusting for total brain volume in DTI analysis to minimize the potential confounding of partial volume effects. Alpha was set at 0.05 for all analyses and the results were corrected for multiple comparisons using the false discovery rate (FDR). All statistical analyses were performed using Stata SE 16.0 (Stata Corp, College Station, Texas; “reg,” “mixed”) and R (version 4.1.1; “cfa”).

## Results

### Baseline characteristics

Among the 37 164 participants included in the cross-sectional analysis, the mean age was 54.86 years (SD = 7.53) and 19 880 (53.5%) were women. Among all participants, the time interval between study entry and first/second MRI assessment was 8.94 ± 1.78/11.05 ± 1.01 years. Compared to participants with high lung function, those with low lung function were more likely to be older, non-white, not college educated, have higher TDI and BMI, have poorer brain health (smaller volumes and poorer white matter health), be a former or current smoker, be non-drinking, have irregular physical activity, have high level of social connection, be an *APOE* ε4 non-carrier, and have hypertension, diabetes, or heart disease (Table 1).

**Table 1:** Characteristics of the study population (N = 37 164) by tertiles of lung function

Characteristics	Lung function*			*P* value
	Low (n = 12 375)	Moderate (n = 12 396)	High (n = 12 393)	
Age (y)	58.10 ± 6.92	55.06 ± 7.09	51.27 ± 6.94	<0.001
Female	6623 (53.52)	6624 (53.44)	6633 (53.52)	-
Race-White	11 262(91.29)	11 522(93.18)	11 529(93.18)	<0.001
Townsend deprivation index	−2.55 (−3.84, −0.31)	−2.66 (−3.92, −0.58)	−2.63 (−3.90, −0.57)	<0.001
Education (college)	5258(42.70)	5874(47.50)	6434(52.00)	<0.001
Body mass index (kg/m^2^)	27.07 ± 4.44	26.45 ± 4.16	25.94 ± 3.89	<0.001
Underweight	272 (2.20)	306 (2.47)	372 (3.00)	<0.001
Normal weight	4012 (32.45)	4702 (37.94)	5267 (42.51)	
Overweight	5430 (43.92)	5265 (42.48)	5109 (41.23)	
Obesity	2649 (21.43)	2120 (17.11)	1643 (13.26)	
Alcohol consumption status				<0.001
Never	433 (3.50)	262 (2.11)	232 (1.87)	
Former drinking	287 (2.32)	246 (1.99)	213 (1.72)	
Current drinking	11 649 (94.18)	11 884 (95.90)	11 944 (96.41)	
Smoking status				<0.001
Never	7440 (60.27)	7524 (60.79)	7911 (63.94)	
Former smoker	4102 (33.23)	4131 (33.38)	3769 (30.46)	
Current smoker	802 (6.50)	722 (5.83)	693 (5.60)	
Passive smoker	3532 (28.54)	3148 (25.40)	2889 (23.31)	<0.001
Regular physical activity	8528 (72.38)	8928 (74.54)	9332 (76.97)	<0.001
High level of social connection	5197 (42.00)	4875 (39.33)	4491 (36.24)	<0.001
Hypertension	6665 (53.86)	5480 (44.21)	4304 (34.73)	<0.001
Diabetes	531 (4.29)	281 (2.27)	175 (1.41)	<0.001
Heart disease	529 (4.27)	280 (2.26)	155 (1.25)	<0.001
*APOE* ε4 carrier	2698 (26.66)	2963 (27.92)	2975 (28.57)	0.008
Brain MRI				
Total brain volume (mm^3^)	1.13 × 10^6^ ± 1.10 × 10^5^	1.16 × 10^6^ ± 1.07 × 10^5^	1.19 × 10^6^ ± 1.10 × 10^5^	<0.001
White matter volume (mm^3^)	5.35 × 10^5^ ± 0.62 × 10^5^	5.46 × 10^5^ ± 0.60 × 10^5^	5.56 × 10^5^ ± 0.61 × 10^5^	<0.001
Grey matter volume (mm^3^)	5.97 × 10^5^ ± 0.55 × 10^5^	6.15 × 10^5^ ± 0.53 × 10^5^	6.33 × 10^5^ ± 0.54 × 10^5^	<0.001
Hippocampus volume (mm^3^)	7.48 ± 0.87	7.67 ± 0.86	7.85 ± 0.88	<0.001
WMH (mm^3^)^a^	8.24 ± 0.98	8.01 ± 0.98	7.72 ± 0.97	<0.001
gFA normalized	−0.11 (−0.83, 0.54)	0.06 (−0.62, 0.70)	0.21 (−0.46, 0.85)	<0.001
gMD normalized	0.12 (−0.55, 0.85)	−0.10 (−0.70, 0.59)	−0.28 (−0.83, 0.34)	<0.001

Abbreviations: *APOE* ε4 = apolipoprotein E epsilon; BMI = body mass index; WMH = white matter hyperintensity; gFA and gMD (latent factors of white matter fractional anisotropy and mean diffusivity).

Missing data: education = 156; race = 93; TDI = 37; smoking = 73; alcohol consumption = 16; BMI = 22; regular physical activity = 1,328; social connection = 144; *APOE* ε4 genotype = 6,166.

Data was presented as mean ± standard deviation or median (p_25_, p_75_) for continuous variables and number (percentage) for categorical variables.

* Lung function was classified by sex-specified tertile: tertile 1 (lung function ≤ −0.81); tertile 2 (−0.81 to −0.32); tertile 3 (>−0.32) for female; and tertile 1 (lung function ≤ 0.32); tertile 2 (0.32 to 0.99); tertile 3 (>0.99) for male.

^a^ The volume of white matter hyperintensities was transformed by taking the logarithm.

Compared to the participants who only underwent the first MRI assessment, those who underwent a second MRI scan were more likely to be younger, white, have regular physical activity, have higher lung function and BMI, be an *APOE* ε4 non-carrier, and be diabetes- and hypertension-free (Supplementary Table 4, available at: http://links.lww.com/JS9/D766).

### Cross-sectional association between lung function and structural brain differences

Cross-sectionally, better lung function (as a continuous variable) was related to larger volume of total brain, white matter, gray matter, and hippocampus, as well as smaller WMH volume (Table 2). As a categorical variable, moderate/low lung function, compared to high lung function, was related to smaller total brain, gray matter, white matter, and hippocampal volumes but larger WMH volume (Table 2). Lung function was significantly associated with most of regional gray matter volumes, and the associations were generally similar across different regions (Supplementary Table 5, available at: http://links.lww.com/JS9/D766). Better lung function (as a continuous variable) was also associated with poorer white matter health across all DTI parameters (Table 3). Compared to high lung function, low lung function was related to lower FA and higher MD in global, association fibers, commissural, and projection fibers (Table 3). Similar results were found in relation to the three individual lung function indicators (i.e., FEV1, FVC, and PEF) as well as FEV1/FVC (Supplementary Table 6, available at: http://links.lww.com/JS9/D766).

**Table 2:** Association between lung function and total and structural brain volumes

Composite lung function	Total brain	Gray matter	White matter	Hippocampus	WMH
	β (95% CI)*	β (95% CI)*	β (95% CI)*	β (95% CI)*	β (95% CI)*
**Cross-sectional analysis**					
Continuous	0.048^a^	0.085^a^	0.009^a^	0.061^a^	−0.160^a^
	(0.040 to 0.055)	(0.076 to 0.094)	(0.001 to 0.018)	(0.045 to 0.076)	(−0.175 to −0.145)
Categories					
High	**Reference**	**Reference**	**Reference**	**Reference**	**Reference**
Moderate	−0.053^a^	−0.089^a^	−0.015^a^	−0.050^a^	0.165^a^
	(−0.065 to −0.040)	(−0.103 to −0.074)	(−0.029 to −0.001)	(−0.075 to −0.026)	(0.141 to 0.189)
Low	−0.077^a^	−0.136^a^	−0.017^a^	−0.096^a^	0.266^a^
	(−0.091 to −0.064)	(−0.151 to −0.120)	(−0.032 to −0.002)	(−0.123 to −0.069)	(0.240 to 0.292)
**Longitudinal analysis (lung function × time)**					
Continuous	0.004	0.004	0.008	0.008	−0.008
	(−0.001 to 0.009)	(−0.002 to 0.011)	(0.000 to 0.016)	(−0.005 to 0.021)	(−0.028 to 0.012)
Categories					
High	**Reference**	**Reference**	**Reference**	**Reference**	**Reference**
Moderate	−0.008	0.005	−0.021	−0.016	0.053
	(−0.019 to −0.003)	(−0.011 to 0.021)	(−0.040 to −0.003)	(−0.046 to 0.015)	(0.007 to 0.100)
Low	−0.011	−0.004	−0.021^a^	−0.076^a^	0.066^a^
	(−0.023 to 0.001)	(−0.021 to 0.013)	(−0.041 to −0.001)	(−0.108 to −0.043)	(0.016 to 0.115)

Abbreviations: WMH = white matter hyperintensity.

All MRI measures were Z-transformed in order to minimize errors due to variance variation and inconsistency in the units of the variables, which resulted in relatively small β coefficients (indicating differences in Z-scores rather than the raw data).

* Model adjusted for age, sex, education, race, Townsend deprivation index, body mass index, alcohol consumption, smoking, physical activity, social connection, hypertension, diabetes, heart disease, *APOE* ε4, and head position MRI confounds (volumetric data are also corrected for head size). Standardized coefficients (β) and confidence intervals (CI) are reported from regression models.

^a^ FDR q < 0.05.

**Table 3:** Association between lung function and white matter micro-structure parameters

Composite lung function	Fractional anisotropy (FA)				Mean diffusivity (MD)			
	gFA	Association fibers	Commissural fibers	Projection fibers	gMD	Association fibers	Commissural fibers	Projection fibers
	β (95% CI)*	β (95% CI)*	β (95% CI)*	β (95% CI)*	β (95% CI)*	β (95% CI)*	β (95% CI)*	β (95% CI)*
**Cross-sectional analysis**								
Continuous	0.096^a^	0.092^a^	0.111^a^	0.047^a^	−0.095^a^	−0.083^a^	−0.130^a^	−0.045^a^
	(0.078 to 0.113)	(0.074 to 0.109)	(0.094 to 0.129)	(0.030 to 0.065)	(−0.112 to −0.079)	(−0.100 to −0.066)	(−0.146 to −0.115)	(−0.062 to −0.027)
Categories								
High	**Reference**	**Reference**	**Reference**	**Reference**	**Reference**	**Reference**	**Reference**	**Reference**
Moderate	−0.089^a^	−0.090^a^	−0.101	−0.042^a^	0.089^a^	0.081^a^	0.118^a^	0.025
	(−0.117 to −0.061)	(−0.119 to −0.062)	(−0.129 to −0.074)	(−0.070 to −0.014)	(0.062 to 0.115)	(0.054 to 0.108)	(0.093 to 0.143)	(−0.002 to 0.053)
Low	−0.164^a^	−0.157^a^	−0.188^a^	−0.105^a^	0.168^a^	0.148^a^	0.224^a^	0.075^a^
	(−0.194 to −0.134)	(−0.187 to −0.127)	(−0.218 to −0.158)	(−0.135 to −0.075)	(0.139 to 0.196)	(0.119 to 0.177)	(0.197 to 0.250)	(0.045 to 0.105)
**Longitudinal analysis (lung function × time)**								
Continuous	0.003	0.004	0.008	0.026	−0.030^a^	−0.026^a^	−0.031^a^	−0.061^a^
	(−0.008 to 0.014)	(−0.008 to 0.015)	(−0.006 to 0.022)	(0.008 to 0.045)	(−0.043 to −0.017)	(−0.039 to −0.014)	(−0.045 to −0.016)	(−0.085 to −0.038)
Categories								
High	**Reference**	**Reference**	**Reference**	**Reference**	**Reference**	**Reference**	**Reference**	**Reference**
Moderate	−0.025	−0.023	−0.030	−0.010	0.057^a^	0.050^a^	0.068^a^	0.056^a^
	(−0.051 to 0.001)	(−0.050 to 0.004)	(−0.063 to 0.002)	(−0.054 to 0.034)	(0.026 to 0.087)	(0.021 to 0.080)	(0.033 to 0.102)	(0.002 to 0.110)
Low	−0.058^a^	−0.059^a^	−0.042^a^	0.001	0.118^a^	0.101^a^	0.133^a^	0.139^a^
	(−0.086 to −0.030)	(−0.088 to −0.030)	(−0.077 to −0.008)	(−0.046 to 0.048)	(0.085 to 0.151)	(0.069 to 0.132)	(0.097 to 0.170)	(0.081 to 0.197)

Abbreviations: gFA and gMD (latent factors of white matter fractional anisotropy and mean diffusivity).

* Model adjusted for age, sex, education, race, Townsend deprivation index, body mass index, alcohol consumption, smoking, physical activity, social connection, hypertension, diabetes, heart disease, *APOE* ε4, and head position MRI confounds (volumetric data are also corrected for head size). Standardized coefficients (β) and confidence intervals (CI) are reported from regression models.

^a^ FDR q < 0.05.

### Longitudinal association of lung function with brain aging

Longitudinally, compared to high lung function, low lung function was related to a loss of white matter and hippocampal volume and an increase in WMH volume, but not with changes in total brain or gray matter volume (Table 2). However, as a continuous variable, lung function was not associated with changes in any volumetric brain MRI parameters (Table 2).

In the second MRI assessment analysis, better lung function (as a continuous variable) was related to fewer changes in MD including global, association fibers, and commissural fibers (Table 3). Compared to the high lung function, low lung function was related to faster decrease in FA including global, association fibers, and commissural fibers, and greater increase in MD including global, association fibers, commissural fibers, and projection fibers (Table 3). Regression coefficients for the covariates in relation to brain volumes are shown in Supplementary Table 7, available at: http://links.lww.com/JS9/D766, and the results were analyzed by the multivariable adjusted regression model (i.e., lung function and covariates were included in the same model together). The other fixed effects (including lung function and follow-up time) and random effects in the linear mixed-effects models are shown in Supplementary Table 8, available at: http://links.lww.com/JS9/D766.

### Lung function-brain aging association in mid- and late-life

**Figure 2.**
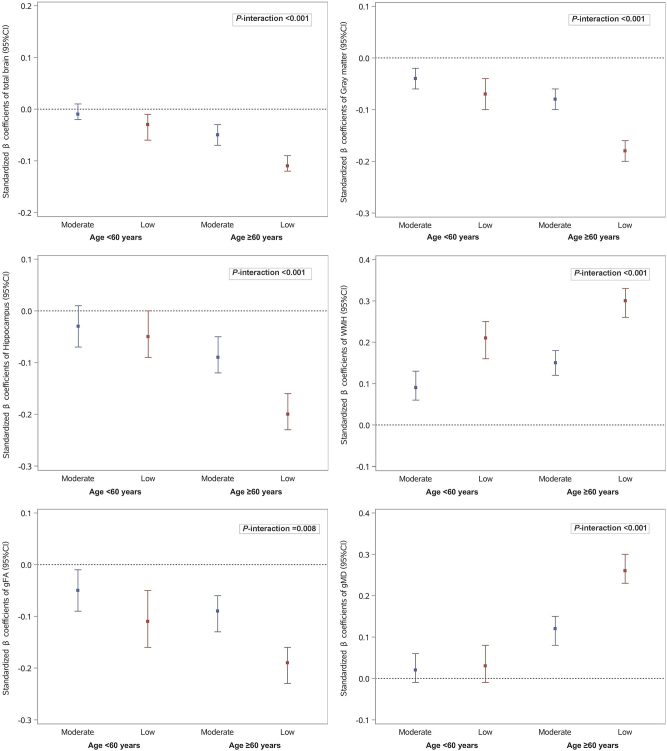
Association between lung function and brain magnetic resonance imaging (MRI) parameters: stratified by age group (reference: high lung function).

In stratified analysis by age, the association between poor lung function and brain aging remained significant even in mid-life (<60 years), although this association was stronger in late life (≥60 years). The interaction between lung function and age on total brain, gray matter, hippocampus, WMH, gFA, and gMD was statistically significant in the cross-sectional data analysis (FDR *P*-interaction <0.05), but not significant in the longitudinal data analysis (FDR *P*-interaction >0.05) (Fig. 2 and Supplementary Table 9, available at: http://links.lww.com/JS9/D766).

### Sensitivity analysis

In sensitivity analysis, similar results to those from the main analyses were obtained after we (1) performed multiple imputations for missing values of some covariates (education, race, TDI, smoking, alcohol consumption, BMI, physical activity, and social connection (Supplementary Table 10, available at: http://links.lww.com/JS9/D766); (2) further adjusted for MRI assessment center (Supplementary Table 11, available at: http://links.lww.com/JS9/D766); (3) and further adjusted for respiratory disease (Supplementary Table 12, available at: http://links.lww.com/JS9/D766); (4) using FEV1% predicted as lung function indicator (Supplementary Table 13, available at: http://links.lww.com/JS9/D766); (5) further adjusted for smoking pack years (Supplementary Table 14, available at: http://links.lww.com/JS9/D766); and (6) further adjusting for total brain volume in DTI analysis (Supplementary Table 15, available at: http://links.lww.com/JS9/D766).

## Discussion

In this large brain imaging sample of mid- and late-life adults, we found that individuals with lower lung function have smaller total brain, white matter, grey matter, and hippocampal volume, as well as poorer white matter health, including greater WMH load, poorer FA, and higher MD. In addition, low lung function was associated with faster white matter and hippocampal atrophy, faster decline in FA, and greater increase in MD and WMH volume. Moreover, these associations were present in both mid- and late-life.

Several longitudinal studies have linked decreased lung function to cognitive decline and an increased risk of dementia^[6,7,10,22,23,34]^, but only a few studies have addressed the association between lung function and brain MRI measures, with inconsistent findings^[7,16-19]^. Within the Rush Memory and Aging Project, we showed that poor lung function (assessed using a composite measure including FEV1, FVC, and PEF) was associated with smaller total brain, white matter, and gray matter volumes, and greater WMH, but no difference in hippocampal volume^[7]^. A recent study from the UK Biobank reported cross-sectional associations between restrictive and obstructive injuries and brain MRI measures including lower total brain volume, greater WMH, poorer white matter integrity, and smaller hippocampal volume^[19]^. A study from ARIC Neurocognitive Study showed that FEV1 and FVC were cross-sectionally associated with total brain and WMH volumes^[35]^. Two longitudinal studies have indicated that FVC% (but not FEV1% predicted or FEV1/FVC) was related to WMH load^[14,15]^, but not other brain MRI measures. In contrast, another cohort study reported that neither FEV1, FVC, nor FEV1/FVC were associated with overall brain atrophy or hippocampal or WMH volume^[18]^. Moreover, several studies have also shown that poor lung function is associated with older brain age based on neuroimaging data^[36,37]^. A Mendelian Randomization study reported that FVC, but not FEV1 or FEV1/FVC, was causally related to global surficial area but not to cortical thickness^[38]^. However, most of these studies are cross-sectional with limited sample sizes and without comprehensive measures of lung function. A composite measure including FEV1, FVC, and PEF could yield a more stable measure of lung function and increase statistical power to identify lung dysfunction as well as adverse health consequences of lung dysfunction^[26]^. Questions remain regarding the extent to which poor lung function may affect macro- and micro-brain aging over time. In the present study, we assessed multiple dimensions of lung function and found that composite lung function as well as each individual lung function index (i.e., FEV1, FVC, PEF, and FEV1/FVC) were associated with total and structural brain volumes including gray matter, white matter, hippocampus, and WMH (cerebral white matter microvascular lesions). In particular, low lung function was associated with white matter damage (including faster white matter atrophy, faster decline in FA, and greater increase in MD and WMH volume) and hippocampal atrophy, but not with gray matter atrophy. Our findings also suggest that lung function is more strongly associated with white matter lesions than other structural brain volumes, which may indicate that white matter is more vulnerable to hypoxia and hypoperfusion.

White matter microstructural indicators, including FA and MD, appear to be the most susceptible to aging^[33]^. However, these measures, obtained by diffusion MRI (dMRI), are difficult to collect on a large population scale, as they require participants to remain still for an extended period of time. Two epidemiological studies with restricted samples reported an inverse relationship between FEV1 and MD among people with COPD^[39,40]^. Our study is one of only a few investigations to systematically explore the longitudinal association between lung function (assessed with a composite lung function measure) and macro- and micro-structural changes in the brain using a large-scale population-based study. We found that decreased lung function was associated with poor white matter microstructure (higher MD and lower FA), across global, association fibers, commissural fibers, and projection fibers. This suggests that low lung function may be extensively associated with white matter microstructural integrity, which may be an important pathway underlying lung function-brain aging association.

Both lung function decline and changes in brain structure are strongly age-related^[20,21]^. Evidence has shown that lung function in middle age is also related to cognitive status in later life^[22,23]^. Middle-aged and older adults have different characteristics in various aspects of physical functioning and health status. However, comprehensive investigations into the influence of lung function on brain structure at different ages remain limited, but could provide insights into effective prediction and targeted prevention of brain aging. In particular, it is unclear whether the association between lung function and brain health observed in older adults is also present in middle-age. In the current study, we found that the association between poor lung function and brain atrophy was present not only in older adults but also in middle age. Our findings suggest that monitoring brain aging related to lung function is relevant already in mid-life.

Several possible mechanisms could link poor lung function to brain aging. First, poor lung function may affect the supply of oxygen to the brain (which consumes nearly 20% of the body’s oxygen), making the brain, especially the white matter, more susceptible to hypoxia^[41]^. Cerebral hypoxia may cause vascular endothelial dysfunction or enhancement of blood clotting activity, which are leading causes of cerebral ischemic lesions^[42]^, in which chronic systemic inflammation, tissue hypoxia, and oxidative stress play crucial roles^[42,43]^. Second, poor lung function is associated with a higher risk of cardiovascular events^[44]^, which may lead to poor brain health by accelerating vascular damage and degenerative lesions^[45]^. Third, individuals with poor lung function might incline to less physical activity and physical function^[46]^, which in turn is linked to less brain integrity^[47]^. Finally, decreased lung function and brain aging are both concomitant changes in the aging process caused by the accumulation of diverse deleterious changes in the cells and tissues with advancing age^[48]^. Future longitudinal studies on the mediating role of cerebral hypoxia and inflammation in the lung function-brain health association are warranted to better understand the underlying mechanisms^[49,50]^.

Strengths of this study include the large-scale community-based design with a large sample size and a comprehensive data collection procedure. Additionally, the UK Biobank provides image-derived phenotypes of various brain measures, contributing to the understanding of the integrative association between lung function and brain health. Nonetheless, some limitations should be pointed out. First, the UK Biobank participants were volunteers likely healthier than the general population. This might have contributed to an underestimation of the magnitude of the association between poor lung function and brain aging. Furthermore, caution is required when generalizing our results to populations outside of white European ancestry. Second, brain MRI assessment was conducted at different centers, which might lead to measurement bias. However, our results were not meaningfully impacted after repeating the analysis with assessment center as an additional cofounder. Third, this study tested the longitudinal association of poor lung function with MRI measures collected 9–12 years later and excluded participants with self-reported chronic brain disorders. However, only a small sample underwent two MRI scans, resulting in a large difference in sample size between the cross-sectional and longitudinal analyses. Fourth, data on other lung parameters (lung perfusion) and brain blood flow signals were not available and therefore could not be included in the current analysis. Fifth, although the composite lung function has been validated in our previous studies using the MAP population, it has not been used in other study populations. However, results were similar after repeating the analysis using individual lung function measures (FEV1, FVC, or PEF). Further population-based longitudinal studies using the composite score and Mendelian Randomization study are warranted. Sixth, since lung function was measured at baseline, there may be a reverse causality between brain structure and lung function. However, we addressed this by excluding participants with baseline chronic brain disorder or chronic obstructive pulmonary disease, and the lung function-brain structure association remained significant. Thus, the temporality of the observed association is clear. Finally, selection bias might have occurred due to missing data. However, after repeating the analysis using multiple imputations for missing variables, the results were not much altered compared to those from the initial analysis.

## Conclusion

In conclusion, our study provides evidence that low lung function is associated with brain aging at both macro- and micro-structural levels possibly involving neurodegenerative and vascular pathologies in mid- and late-life. Our findings underscore the importance of maintaining lung health to promote brain health in mid- and late-life alike.

## Data Availability

The datasets generated and analyzed during the current study are available in the UK Biobank repository, http://www.ukbiobank.ac.uk.

## Research registration unique identifying number (UIN)

Ref. 11/NW/0382.

## Presentation

Part of the results has been presented as a poster at the Alzheimer’s Association International Conference 2023.
